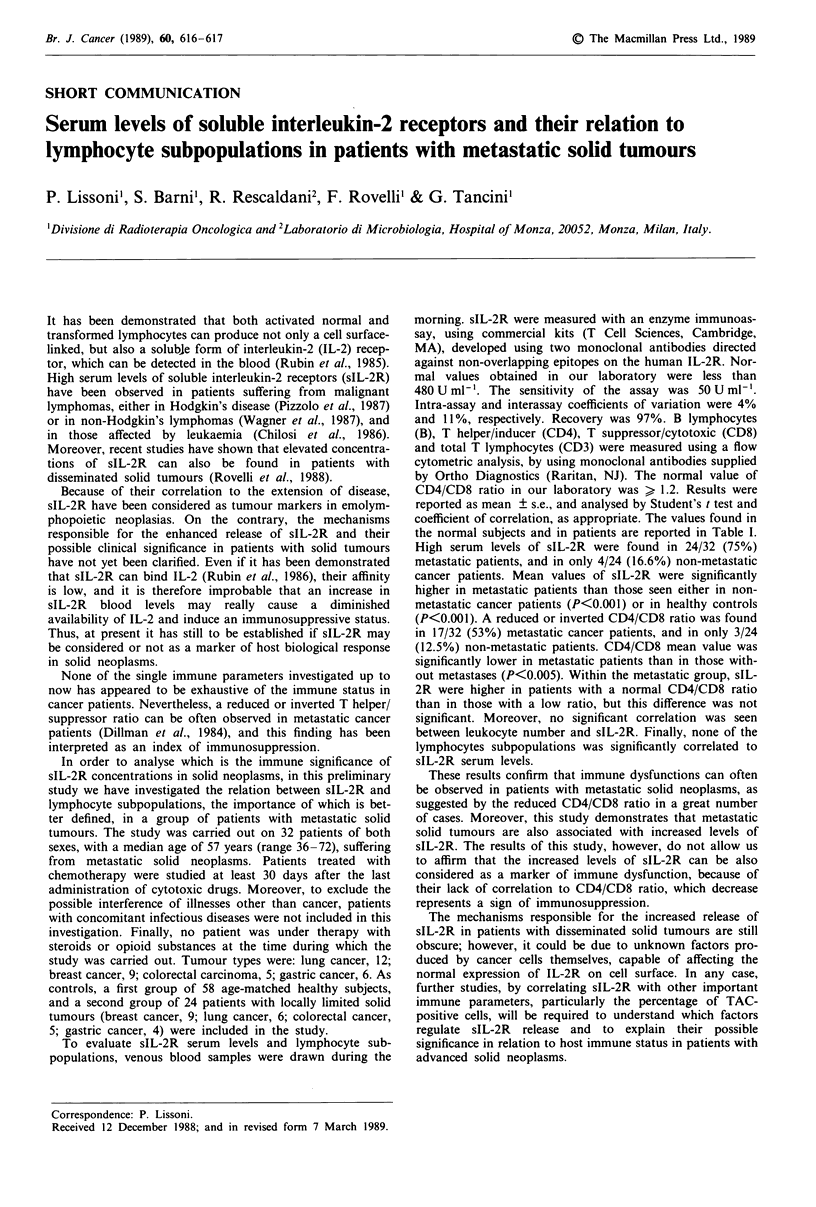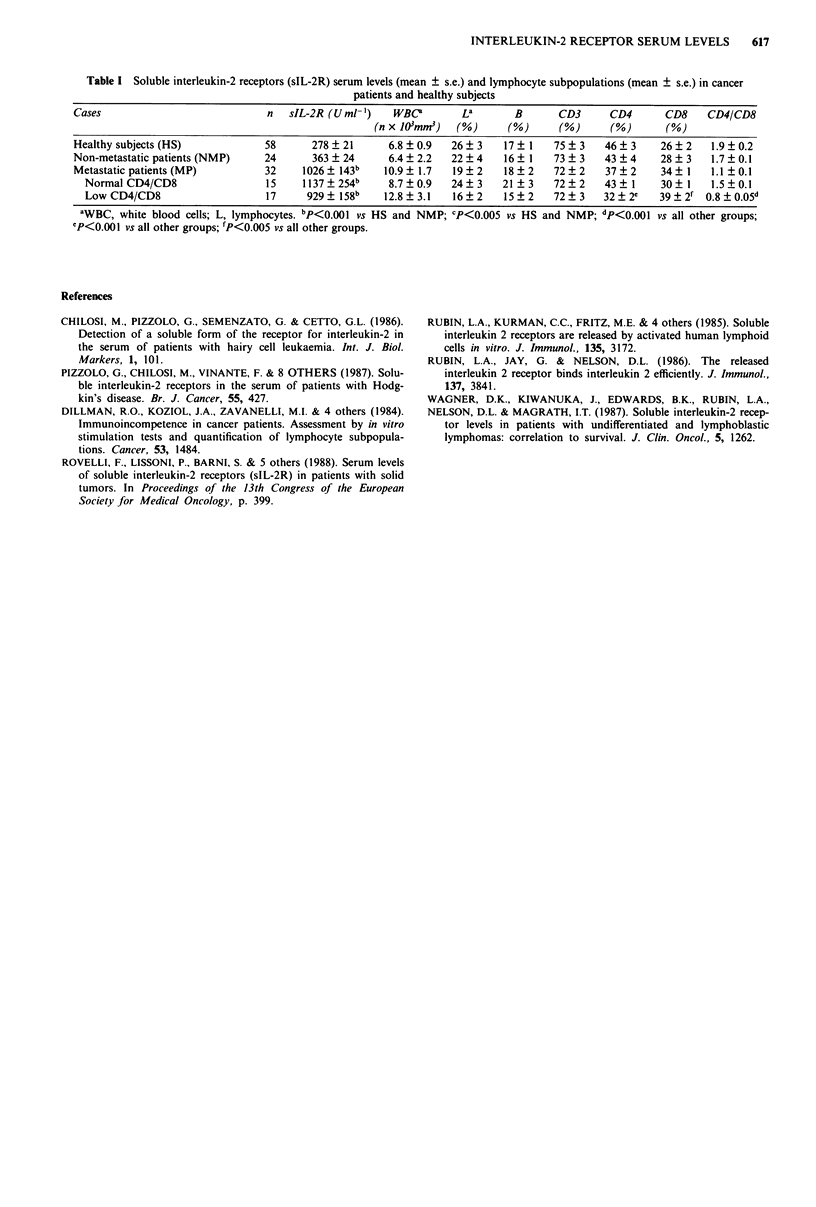# Serum levels of soluble interleukin-2 receptors and their relation to lymphocyte subpopulations in patients with metastatic solid tumours.

**DOI:** 10.1038/bjc.1989.325

**Published:** 1989-10

**Authors:** P. Lissoni, S. Barni, R. Rescaldani, F. Rovelli, G. Tancini

**Affiliations:** Divisione di Radioterapia Oncologica, Hospital of Monza, Milan, Italy.


Br. J. Cancer (1989), 60, 616-617 © The Macmillan Press Ltd., 1989

SHORT COMMUNICATION

### Serum levels of soluble interleukin-2 receptors and their relation to lymphocyte subpopulations in patients with metastatic solid tumours

P. Lissoni[1], S. Barni[1], R. Rescaldani[2], F. Rovelli[1] & G. Tancini[1]

[1]*Divisione di Radioterapia Oncologica and* [2]*Laboratorio di Microbiologia, Hospital of Monza, 20052, Monza, Milan, Italy.*

It has been demonstrated that both activated normal and transformed lymphocytes can produce not only a cell surface-linked, but also a soluble form of interleukin-2 (IL-2) receptor, which can be detected in the blood (Rubin *et al.*, 1985). High serum levels of soluble interleukin-2 receptors (sIL-2R) have been observed in patients suffering from malignant lymphomas, either in Hodgkin's disease (Pizzolo *et al.*, 1987) or in non-Hodgkin's lymphomas (Wagner *et al.*, 1987), and in those affected by leukaemia (Chilosi *et al.*, 1986). Moreover, recent studies have shown that elevated concentrations of sIL-2R can also be found in patients with disseminated solid tumours (Rovelli *et al.*, 1988).

Because of their correlation to the extension of disease, sIL-2R have been considered as tumour markers in emolymphopoietic neoplasias. On the contrary, the mechanisms responsible for the enhanced release of sIL-2R and their possible clinical significance in patients with solid tumours have not yet been clarified. Even if it has been demonstrated that sIL-2R can bind IL-2 (Rubin *et al.*, 1986), their affinity is low, and it is therefore improbable that an increase in sIL-2R blood levels may really cause a diminished availability of IL-2 and induce an immunosuppressive status. Thus, at present it has still to be established if sIL-2R may be considered or not as a marker of host biological response in solid neoplasms.

None of the single immune parameters investigated up to now has appeared to be exhaustive of the immune status in cancer patients. Nevertheless, a reduced or inverted T helper/suppressor ratio can be often observed in metastatic cancer patients (Dillman *et al.*, 1984), and this finding has been interpreted as an index of immunosuppression.

In order to analyse which is the immune significance of sIL-2R concentrations in solid neoplasms, in this preliminary study we have investigated the relation between sIL-2R and lymphocyte subpopulations, the importance of which is better defined, in a group of patients with metastatic solid tumours. The study was carried out on 32 patients of both sexes, with a median age of 57 years (range 36–72), suffering from metastatic solid neoplasms. Patients treated with chemotherapy were studied at least 30 days after the last administration of cytotoxic drugs. Moreover, to exclude the possible interference of illnesses other than cancer, patients with concomitant infectious diseases were not included in this investigation. Finally, no patient was under therapy with steroids or opioid substances at the time during which the study was carried out. Tumour types were: lung cancer, 12; breast cancer, 9; colorectal carcinoma, 5; gastric cancer, 6. As controls, a first group of 58 age-matched healthy subjects, and a second group of 24 patients with locally limited solid tumours (breast cancer, 9; lung cancer, 6; colorectal cancer, 5; gastric cancer, 4) were included in the study.

To evaluate sIL-2R serum levels and lymphocyte subpopulations, venous blood samples were drawn during the morning. sIL-2R were measured with an enzyme immunoassay, using commercial kits (T Cell Sciences, Cambridge, MA), developed using two monoclonal antibodies directed against non-overlapping epitopes on the human IL-2R. Normal values obtained in our laboratory were less than $480\,U\,ml^{-1}$. The sensitivity of the assay was $50\,U\,ml^{-1}$. Intra-assay and interassay coefficients of variation were 4% and 11%, respectively. Recovery was 97%. B lymphocytes (B), T helper/inducer (CD4), T suppressor/cytotoxic (CD8) and total T lymphocytes (CD3) were measured using a flow cytometric analysis, by using monoclonal antibodies supplied by Ortho Diagnostics (Raritan, NJ). The normal value of CD4/CD8 ratio in our laboratory was $\geqslant 1.2$. Results were reported as mean $\pm$ s.e., and analysed by Student's $t$ test and coefficient of correlation, as appropriate. The values found in the normal subjects and in patients are reported in Table I. High serum levels of sIL-2R were found in 24/32 (75%) metastatic patients, and in only 4/24 (16.6%) non-metastatic cancer patients. Mean values of sIL-2R were significantly higher in metastatic patients than those seen either in non-metastatic cancer patients ($P<0.001$) or in healthy controls ($P<0.001$). A reduced or inverted CD4/CD8 ratio was found in 17/32 (53%) metastatic cancer patients, and in only 3/24 (12.5%) non-metastatic patients. CD4/CD8 mean value was significantly lower in metastatic patients than in those without metastases ($P<0.005$). Within the metastatic group, sIL-2R were higher in patients with a normal CD4/CD8 ratio than in those with a low ratio, but this difference was not significant. Moreover, no significant correlation was seen between leukocyte number and sIL-2R. Finally, none of the lymphocytes subpopulations was significantly correlated to sIL-2R serum levels.

These results confirm that immune dysfunctions can often be observed in patients with metastatic solid neoplasms, as suggested by the reduced CD4/CD8 ratio in a great number of cases. Moreover, this study demonstrates that metastatic solid tumours are also associated with increased levels of sIL-2R. The results of this study, however, do not allow us to affirm that the increased levels of sIL-2R can be also considered as a marker of immune dysfunction, because of their lack of correlation to CD4/CD8 ratio, which decrease represents a sign of immunosuppression.

The mechanisms responsible for the increased release of sIL-2R in patients with disseminated solid tumours are still obscure; however, it could be due to unknown factors produced by cancer cells themselves, capable of affecting the normal expression of IL-2R on cell surface. In any case, further studies, by correlating sIL-2R with other important immune parameters, particularly the percentage of TAC-positive cells, will be required to understand which factors regulate sIL-2R release and to explain their possible significance in relation to host immune status in patients with advanced solid neoplasms.

Correspondence: P. Lissoni.
Received 12 December 1988; and in revised form 7 March 1989.

**Table I** Soluble interleukin-2 receptors (sIL-2R) serum levels (mean ± s.e.) and lymphocyte subpopulations (mean ± s.e.) in cancer patients and healthy subjects

| *Cases* | *n* | *sIL-2R* ($U\ ml^{-1}$) | *WBC*[a] ($n \times 10^3 mm^3$) | *L*[a] (%) | *B* (%) | *CD3* (%) | *CD4* (%) | *CD8* (%) | *CD4/CD8* |
|---|---|---|---|---|---|---|---|---|---|
| Healthy subjects (HS) | 58 | 278 ± 21 | 6.8 ± 0.9 | 26 ± 3 | 17 ± 1 | 75 ± 3 | 46 ± 3 | 26 ± 2 | 1.9 ± 0.2 |
| Non-metastatic patients (NMP) | 24 | 363 ± 24 | 6.4 ± 2.2 | 22 ± 4 | 16 ± 1 | 73 ± 3 | 43 ± 4 | 28 ± 3 | 1.7 ± 0.1 |
| Metastatic patients (MP) | 32 | 1026 ± 143[b] | 10.9 ± 1.7 | 19 ± 2 | 18 ± 2 | 72 ± 2 | 37 ± 2 | 34 ± 1 | 1.1 ± 0.1 |
| Normal CD4/CD8 | 15 | 1137 ± 254[b] | 8.7 ± 0.9 | 24 ± 3 | 21 ± 3 | 72 ± 2 | 43 ± 1 | 30 ± 1 | 1.5 ± 0.1 |
| Low CD4/CD8 | 17 | 929 ± 158[b] | 12.8 ± 3.1 | 16 ± 2 | 15 ± 2 | 72 ± 3 | 32 ± 2[e] | 39 ± 2[f] | 0.8 ± 0.05[d] |

[a]WBC, white blood cells; L, lymphocytes. [b]$P<0.001$ *vs* HS and NMP; [c]$P<0.005$ *vs* HS and NMP; [d]$P<0.001$ *vs* all other groups; [e]$P<0.001$ *vs* all other groups; [f]$P<0.005$ *vs* all other groups.

### References

CHILOSI, M., PIZZOLO, G., SEMENZATO, G. & CETTO, G.L. (1986). Detection of a soluble form of the receptor for interleukin-2 in the serum of patients with hairy cell leukaemia. *Int. J. Biol. Markers*, **1**, 101.

PIZZOLO, G., CHILOSI, M., VINANTE, F. & 8 OTHERS (1987). Soluble interleukin-2 receptors in the serum of patients with Hodgkin's disease. *Br. J. Cancer*, **55**, 427.

DILLMAN, R.O., KOZIOL, J.A., ZAVANELLI, M.I. & 4 others (1984). Immunoincompetence in cancer patients. Assessment by *in vitro* stimulation tests and quantification of lymphocyte subpopulations. *Cancer*, **53**, 1484.

ROVELLI, F., LISSONI, P., BARNI, S. & 5 others (1988). Serum levels of soluble interleukin-2 receptors (sIL-2R) in patients with solid tumors. In *Proceedings of the 13th Congress of the European Society for Medical Oncology*, p. 399.

RUBIN, L.A., KURMAN, C.C., FRITZ, M.E. & 4 others (1985). Soluble interleukin 2 receptors are released by activated human lymphoid cells *in vitro*. *J. Immunol.*, **135**, 3172.

RUBIN, L.A., JAY, G. & NELSON, D.L. (1986). The released interleukin 2 receptor binds interleukin 2 efficiently. *J. Immunol.*, **137**, 3841.

WAGNER, D.K., KIWANUKA, J., EDWARDS, B.K., RUBIN, L.A., NELSON, D.L. & MAGRATH, I.T. (1987). Soluble interleukin-2 receptor levels in patients with undifferentiated and lymphoblastic lymphomas: correlation to survival. *J. Clin. Oncol.*, **5**, 1262.